# Thought and language disturbance in bipolar disorder quantified via process-oriented verbal fluency measures

**DOI:** 10.1038/s41598-019-50818-5

**Published:** 2019-10-03

**Authors:** Luisa Weiner, Nadège Doignon-Camus, Gilles Bertschy, Anne Giersch

**Affiliations:** 1 INSERM U1114, Strasbourg, France; 2 Psychiatry Department, University Hospital of Strasbourg, Strasbourg, France; 3 Translational Medicine Federation, University of Strasbourg, Strasbourg, France

**Keywords:** Language, Bipolar disorder

## Abstract

Bipolar disorder (BD) is characterized by speech abnormalities, reflected by symptoms such as pressure of speech in mania and poverty of speech in depression. Here we aimed at investigating speech abnormalities in different episodes of BD, including mixed episodes, via process-oriented measures of verbal fluency performance – i.e., word and error count, semantic and phonological clustering measures, and number of switches–, and their relation to neurocognitive mechanisms and clinical symptoms. 93 patients with BD – i.e., 25 manic, 12 mixed manic, 19 mixed depression, 17 depressed, and 20 euthymic–and 31 healthy controls were administered three verbal fluency tasks – free, letter, semantic–and a clinical and neuropsychological assessment. Compared to depression and euthymia, switching and clustering abnormalities were found in manic and mixed states, mimicking symptoms like flight of ideas. Moreover, the neuropsychological results, as well as the fact that error count did not increase whereas phonological associations did, showed that impaired inhibition abilities and distractibility could not account for the results in patients with manic symptoms. Rather, semantic overactivation in patients with manic symptoms, including mixed depression, may compensate for trait-like deficient semantic retrieval/access found in euthymia.

*“For those who are manic, or those who have a history of mania, words move about in all directions possible, in a three-dimensional ‘soup’, making retrieval more fluid, less predictable.”* Kay Redfield Jamison (2017, p. 279).

## Introduction

Bipolar disorder (BD) is characterized by acute episodes of mania and depression, mixed episodes wherein depressive and manic symptoms co-occur, and periods of partial or full remission, also called ‘euthymic states’. Language disturbances such as speech pressure or poverty are among the main symptoms of acute episodes in BD^1^, and may prevail during periods of remission^2^. While early studies focused on associational fluency as a measure of creativity and thinking style in mania^3–7^, more recent studies have favored the use of verbal fluency tasks (VFT) with more restrained instructions (e.g., starting with a given letter or category) to tackle executive and language impairments mostly in euthymia, i.e., during periods of mood stabilization^8^. Here we investigated language disturbances, by means of both free and restrained VFT across different mood episodes of BD, to determine the contribution of clinical symptoms and executive functioning to word production in BD.

Different kinds of language disturbances have been described during mood episodes of BD. Pressure of speech, with increased rapidity of speech and racing thoughts, is a common symptom of mania, second only to elevated mood^9^. Manic speech has also been characterized as extremely combinatory, shifting quickly from one discourse structure to another, which authors have linked to distractibility and overactivation^10^. Other linguistic features frequently reported during manic episodes include increased verbosity^11^, and clang associations, i.e., associations based on sound rather than on the meaning of words^12^. In contrast, poverty of speech and increased pause times are common in depression, and have been hypothesized to be associated with psychomotor retardation and rumination^11^. In mixed episodes, linguistic features have been understudied, but phenomenological accounts suggest that patients may experience ‘disorganized flight of ideas’^13^, distractibility and ‘crowded thoughts’^14^, pressure and poverty of speech^15^. However, few empirical investigations have specifically addressed these thought and language abnormalities, whether in manic, depressed or mixed states. In particular, from a neurocognitive perspective, it is unclear whether manic speech is related to mechanisms such as semantic overactivation and deficient cognitive control.

VFT are widely used neuropsychological methods for studying language disorders^16^. In these tasks, subjects are instructed to generate words according to specified rules based on phonemic or semantic criteria (‘letter’ and ‘semantic’ fluency, respectively), or in the absence of a specified criterion (free word generation). Although traditionally only the total number of words produced within the allotted time period is considered, VFT are multi-faceted^17,18^. Indeed, it has been long known that semantically related words occur together as part of a burst of responding in recall protocols^19^. Qualitative process-oriented methods evolved based on findings relative to the dynamics of word retrieval in fluency and semantic memory tasks. Word output requires the integrity of both the storage and organization of concepts in lexico-semantic memory, and the ability to retrieve words from memory, thought to rely on executive functioning^17^. These processes underlie two aspects of word output that are responsible for optimal performance: the ability to produce words within semantic or phonological clusters, and the ability to shift to a new category, i.e., clustering and switching respectively^17^.

According to Troyer *et al*.^17^, clustering is defined by the production of words within semantic subcategories in the semantic fluency task (e.g., bird subcategory if the category is “animals”), and phonemic subcategories in the letter fluency task (e.g., words that rhyme). The clustering measure of interest is the cluster size, i.e. the number of words within each cluster. In their processing-oriented scoring procedure, Troyer *et al*.^17^ considered task-consistent clustering, i.e., semantic relatedness in semantic fluency and phonemic relatedness in letter fluency. Switching was operationalized as the ability to shift from a subcategory to another. More recent qualitative scoring procedures have integrated ‘task-discrepant’ clustering, consisting of phonemic relatedness in semantic fluency or semantic relatedness in letter fluency^20^. ‘Task-discrepant’ means that when instructed to retrieve words from a given category (e.g. animals), retrieval might include phonologically-related successive words (e.g., cat and bat). Recent scoring procedures have also integrated a measure of cluster ratio (i.e., the number of clusters/number of words), arguing that mean cluster size is an ambiguous measure, as it reflects both the total number of words and the organization of the verbal output^21^. Hence the combined use of the mean cluster size and the cluster ratio is considered a better index of clustering, as it addresses respectively the integrity of lexico-semantic memory as well as retrieval organization throughout the task^22,23^.

In BD however, only the total word count has been considered. In a recent meta-analysis of VFT in BD, Raucher-Chéné *et al*.^8^ found that performance in letter and semantic VFT was equally reduced in patients with BD. Most studies were conducted during euthymia (30 out of 39 studies), and only one study^24^ included a group of patients in a mixed episode. Importantly, Raucher-Chéné *et al*.^8^ found greater impairment in the semantic (but not letter) VFT in euthymic compared to manic patients. The authors argued that semantic memory dysfunction – i.e., storage and/or functional organization – could explain these results. Moreover, akin to formal thought disorder in schizophrenia, they speculated that the relative “manic advantage” in the semantic VFT was related to an over-activation of the semantic network, which supposedly underlies thought and language disturbances in mania^8^. That is, during manic episodes, the oral production of a given word might lead to faster than usual spreading of activation, hence facilitating the retrieval of more remotely associated words. If such is the case, cluster ratio should be reduced, and switches should be increased in manic and mixed groups compared to controls and euthymic and depressed bipolar groups.

Here we applied a comprehensive process-oriented method in patients with BD in five different mood episodes – i.e., mania, mixed mania, mixed depression, depression, and euthymia. To do so, total word count, but also measures of clustering and switching were calculated in three conditions of VFT – i.e., letter, semantic, and free condition: we calculated semantic and phonological cluster ratios (i.e., number of clusters/number of words), mean cluster sizes, and the raw number of switches. Consistent with earlier studies using associational paradigms^3,4^, we expected word production to be decreased in depressed patients and enhanced in patients with manic symptoms. The idiosyncratic combinatory and associational patterns (e.g., clanging) observed in mania^3,10^ were expected to result in enhanced switches, and, possibly, diminished semantic clustering measures, and increased phonological clustering measures compared to healthy controls, euthymia and depression groups. We were particularly interested in the results of the mixed groups, given that distractibility is also a distinctive feature of mixed states^25^. Since linguistic abnormalities in BD were reported either in free speech or in associational fluency tasks, it was unclear, however, if they would still be observed in restricted conditions of VFT (letter or category). Because subjects have to follow specific retrieval rules, these tasks are considered more effortful^26^. Hence executive impairments and clinical symptoms such as distractibility and/or flight of ideas may result in the production of irrelevant words, i.e., errors. In contrast, if it is overactivation in patients with manic symptoms that subtends the peculiarities of manic speech instead of being the consequence of distractibility and overall executive dysfunction, then more switches should be observed while the production of irrelevant words remains stable in tasks with retrieval rules^27^.

## Results

### Descriptive statistics

With the exception of working memory, performance in executive tasks was diminished in mania, mixed mania and depression compared to healthy controls. See Table 1 for detailed results on the neuropsychological tasks and the self-report questionnaires assessing clinical symptoms, i.e., racing thoughts and rumination, and the p significance level for the group effect.

**Table 1:** Means and standard errors of neuropsychological tasks and self-rated questionnaires in patients and controls.

	Mania	Mixed Mania	Mixed Depression	Depression	Euthymia	Controls	p	post-hoc Tukey test
	n = 25	n = 12	n = 20	n = 17	n = 20	n = 31		
TMT A (seconds)	37.04 (3.06)	41.67 (4.24)	32.61 (3.37)	39.82 (3.56)	37.65 (3.28)	26.71 (2.63)	*0.02*	controls > mixed mania and depression
TMT B (seconds)	100.04 (9.29)	95.58 (12.86)	80.11 (10.22)	100.65 (10.80)	80.8 (9.96)	59.03 (8.0)	<*0.01*	controls > mania and depression
Digit-span (raw score)	14.36 (0.69)	13.08 (0.99)	14.47 (0.79)	14.76 (0.84)	14.65 (0.77)	16.16 (0.62)	*0.14*	/
Hayling test inhibition (seconds)	3.53 (0.40)	3.17 (0.57)	3.48 (0.45)	4.84 (0.48)	3.22 (0.44)	3.12 (0.35)	*0.09*	/
Hayling test inhibition (errors)	7.52 (0.69)	5.75 (0.99)	5.26 (0.79)	6.23 (0.83)	5.30 (0.77)	3.59 (0.62)	<*0.01*	controls > mania
Digit-symbol (raw score)	63.88 (3.45)	57.83 (4.98)	70.67 (3.96)	58.47 (4.18)	69.9 (3.86)	84.39 (3.1)	<*0.01*	controls > clinical groups except mixed depression
Vocabulary (raw score)	36.04 (2.02)	30.50 (2.92)	39.47 (2.32)	37.53 (2.46)	44.8 (2.27	36.77 (1.82)	<*0.01*	euthymia > mixed mania
NART (IQ)	109.68 (1.31)	105.83 (1.90)	109.95 (1.51)	108.35 (1.6)	110.35 (1.47)	113.1 (1.18)	*0.03*	controls > mixed mania
RCTQ total	58.64 (6.04)	99.08 (8.72)	75.42 (6.93)	44.47 (7.33)	11.15 (6.75)	7.29 (5.42)	<*0.001*	
RCTQ overactivation	15.24 ((1.4)	22 (2.02)	16.42 (1.6)	8.94 (1.69)	2.85 (1.56)	2.87 (1.25)	<*0.001*	
RCTQ burden	9.36 (1.35)	19.83 (1.95)	15.63 (1.55)	7.58 (1.64)	1.65 (1.51)	0.13(1.22)	<*0.001*	
RCTQ overexcitability	13.88 (1.48)	22.83 (2.13)	16.68 (1.69)	12.59 (1.79)	3.05 (1.65)	2.16 (1.33)	<*0.001*	
RRS brooding	9.48 (0.63)	14.64 (0.94)	15.26 (0.72)	11.13 (0.81)	8.2 (0.7)	6.77 (0.56)	<*0.001*	

TMT = Trail Making Test; NART = National Adult Reading Test; RCTQ = Racing and Crowded Thoughts Questionnaire; RRS = Rumination Response Scale; significant results are presented in italics.

### Number of words

**Figure 1:**
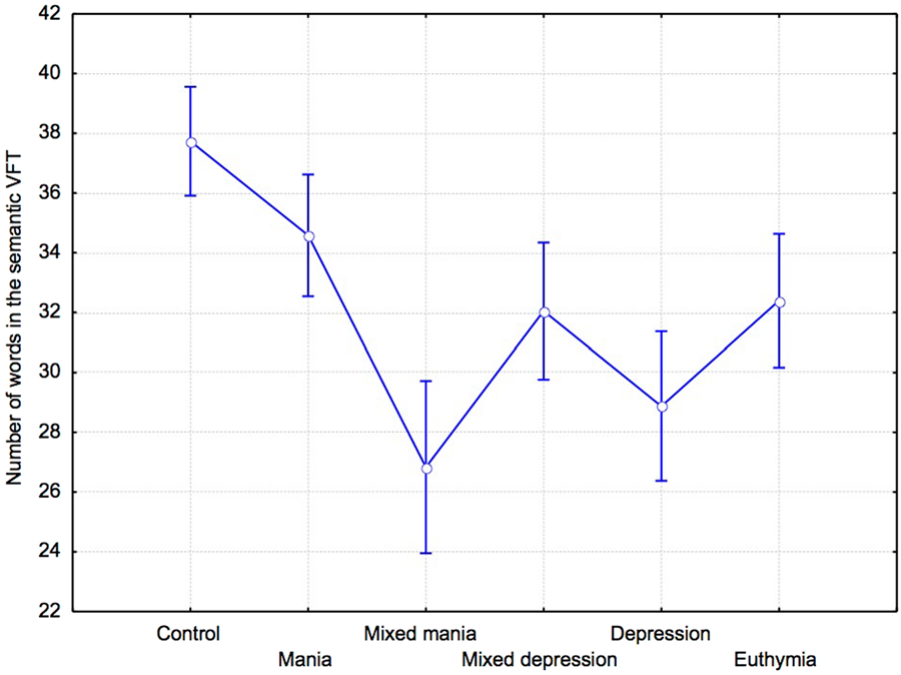
Number of words (mean and standard error) in the semantic VFT.

In the free condition and the letter condition, no significant difference was found in the number of words produced between groups, F(5,118) = 0.45, p = 0.81, η2 = 0.02, and F(5,118) = 0.72, p = 0.60, η2 = 0.04, respectively. In the semantic condition, the number of words produced among groups tended to differ, F(5,118) = 2.14, p = 0.07, η2 = 0.08 (Fig. 1). Planned comparisons revealed that the control and the manic groups tended to produce more animal words than the depressed group, F(1,118) = 4.13, p = 0.08, η2 = 0.1, and F(1,118) = 2.85, p = 0.09, η2 = 0.09, respectively. Number of errors did not differ between groups in the letter and semantic conditions, F(5,117) = 1.37, p = 0.25, η2 = 0.06, and F(5,117) = 0.80, p = 0.55, η2 = 0.03, respectively.

### Cluster analyses

#### Semantic cluster size

Average semantic cluster size did not differ between groups in the free, F(5,118) = 1.4, p = 0.23, η2 = 0.06, the semantic, F(5,118) = 1.4, p = 0.23, η2 = 0.06, and the letter conditions, F(5,117) = 1.56, p = 0.17, η2 = 0.06.

#### Ratio of semantic clusters

The ratio of semantic clusters did not differ between groups in the free, F(5,118) = 1.52, p = 0.19, η2 = 0.06, the letter, F(5,118) = 1.12, p = 0.36, η2 = 0.05, and the semantic conditions, F(5,118) = 0.97 p = 0.44, η2 = 0.42. Nevertheless, in the free condition, planned comparisons revealed that manic groups had significantly smaller cluster ratios than healthy controls, F(1,118) = 4.76, p = 0.03, η2 = 0.09, but not compared to the depressed group, F(1,118) = 0.78, p = 0.77, η2 = 0.02.

#### Phonological cluster size

**Figure 2:**
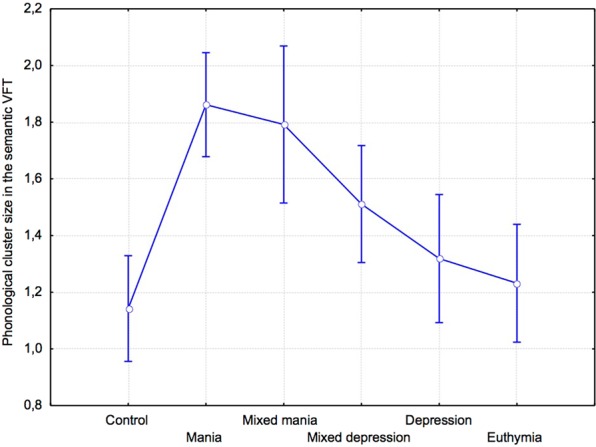
Phonological cluster size (mean and standard error) in the semantic VFT.

Average phonological cluster size did not differ between groups in the free, F(5,118) = 0.94, p = 0.45, η2 = 0.04, and in the letter condition, F(5,117) = 0.35, p = 0.89, η2 = 0.01. In the semantic condition, phonological cluster size tended to differ between groups, F(5,118) = 2.11, p = 0.07, η2 = 0.09 (Fig. 2). As expected, planned comparisons revealed that average phonological cluster size in the semantic condition was significantly increased in the manic compared to the control, F(1,118) = 6.2, p = 0.02, η2 = 0.1, and euthymic groups, F(1,118) = 5.29,p = 0.02, η2 = 0.1. Compared to depressed patients, the difference was only tendential, F(1,118) = 3.86, p = 0.07, η2 = 0.09.

#### Ratio of phonological clusters

The ratio of phonological clusters tended to differ in the free, F(5,118) = 1.91, p = 0.09, η2 = 0.08, and the letter conditions, F(5,117) = 2.3, p = 0.06, η2 = 0.09. In the semantic condition, the ratio of phonological clusters did not differ between groups, F(5,117) = 0.89, p = 0.49, η2 = 0.04.

### Switches

**Figure 3:**
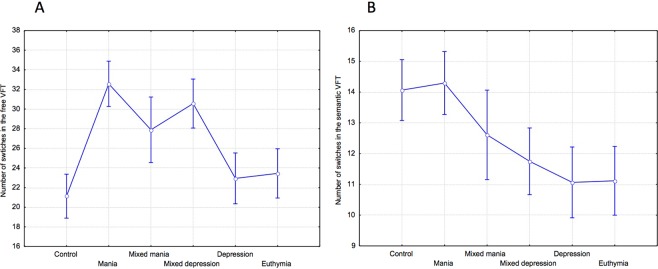
(**A**) Number of switches (mean and standard error) in the free and (**B**) the semantic VFT.

In the free condition, the number of switches differed significantly between groups, F(5,118) = 3.7, p = 0.004, η2 = 0.14 (Fig. 3A). Planned comparisons showed that the number of switches was significantly increased in the manic group compared to the control, F(1,118) = 11.42, p < 0.0001, η2 = 0.26, the depressed groups, F(1,118) = 7.64, p < 0.001, η2 = 0.2, and the euthymic group, F(1,118) = 7.35, p < 0.01, η2 = 0.17. Compared to the depression group, switches were increased in the mixed depression group, F(1,118) = 4.65, p = 0.04, η2 = 0.11. In the letter and semantic conditions, there was no significant difference in the number of switches found among groups, F(5,117) = 0.48, p = 0.79, η2 = 0.02, and F(5,117) = 1.77, p = 0.12, η2 = 0.08, respectively (Fig. 3B). In the semantic condition however, planned comparisons showed that the number of switches was significantly higher in the manic group compared to the depressed and euthymic groups, F(1,117) = 4.37, p = 0.04, η2 = 0.13, and F(1,117) = 4.5, p = 0.04, η2 = 0.13, respectively, but not the control group, F(1,117) = 0.004, p = 0.95, η2 < 0.001.

### Correlation and regression analyses

Correlation analyses were performed within the whole patient group (cf. Table 2). Increased working memory, executive functioning, and processing speed scores were related to greater verbal output in all verbal fluency tasks, whereas increased vocabulary score was only involved in semantic and letter fluency performance. Of note, similar patterns of correlations were found when the sample of patients with manic symptoms (n = 53)–i.e., mania, mixed mania and mixed depression–was considered alone (see Table 4 in supplementary information for detailed results). In addition, to investigate the relationship between process-oriented measures and word output in patients, we performed multiple regression analyses on the number of words produced in the three VFT. For the free condition, predictors accounted for 49% of the variance, with significant contributions from (i) semantic cluster size (β = 0.67, p < 0.001), (ii) ratio of semantic clusters (β = 0.62, p < 0.001), and (iii) number of switches (β = 0.37, p < 0.001). For the semantic condition, the predictors accounted for 50% of the variance, with significant effects of (i) semantic cluster size (β = 0.50, p < 0.001) and (ii) number of switches (β = 0.58, p < 0.001). Regarding the letter condition, the predictors accounted for 76% of the variance, with significant contributions from (i) ratio of phonological clusters (β = 0.36, p < 0.001), and (ii) number of switches (β = 0.89, p < 0.001).

**Table 2:** Correlations between VFT, neuropsychological and clinical measures in patients (n = 90).

Instruments	TMT - A	TMT-B	Digit-span	Hayling time	Hayling errors	Digit-Symbol	Vocabulary	RCTQ	RCTQ	RCTQ	RCTQ	RRS	YMRS	QIDS-C16
								total	overactivation	burden	overexcitability	brooding		
**Number of words**														
Free	−0.28*	−0.25*	0.45*	−0.30*	−0.30*	0.28*	0.12	−0.15	−0.08	−0.17	−0.14	−0.31*	0.09	−0.14
Semantic	−0.30*	−0.34*	0.34*	−0.02	−0.19	0.36*	0.25*	−0.22*	−0.15	−0.20	−0.24*	−0.24*	0.05	−0.06
Letter	−0.19	−0.21	0.32*	−0.04	−0.27*	0.29*	0.23*	−0.23*	−0.20	−0.18	−0.23*	−0.19	−0.15	−0.05
**Semantic cluster ratio**														
Free	−0.08	−0.12	0.09	0.05	0.01	0.07	−0.05	−0.04	0.01	−0.03	−0.06	−0.07	−0.11	−0.01
Semantic	−0.01	−0.01	0.05	0.21*	0.04	0.08	0.02	−0.11	−0.04	−0.08	−0.16	−0.09	0.04	−0.01
Letter	−0.03	0.04	−0.01	0.24*	−0.06	0.04	0.02	−0.13	−0.12	−0.12	−0.12	−0.04	−0.10	0.04
**Semantic cluster size**														
Free	0.14	0.01	−0.07	−0.10	−0.05	−0.05	0.14	−0.11	−0.12	−0.10	−0.09	−0.16	0.02	−0.14
Semantic	−0.01	−0.02	0.07	0.21*	0.02	0.09	0.04	−0.11	−0.04	−0.08	−0.17	−0.09	0.03	−0.01
Letter	−0.02	0.06	−0.01	0.26*	−0.05	0.04	0.03	−0.14	−0.13	−0.13	−0.13	−0.04	−0.11	0.04
**Phonological cluster ratio**														
Free	0.08	0.08	0.15	−0.03	−0.07	−0.06	0.21*	−0.20	−0.13	−0.23*	−0.19	−0.19	0.01	−0.17
Semantic	0.01	0.07	0.02	−0.02	−0.06	0.01	0.05	−0.10	−0.05	−0.14	−0.06	−0.20	0.14	−0.03
Letter	0.21	0.08	0.02	0.05	−0.02	−0.12	0.14	−0.29*	−0.33*	−0.23*	−0.27*	−0.14	−0.22*	−0.01
**Phonological cluster size**														
Free	−0.07	−0.03	0.23*	−0.15	−0.15	0.02	0.03	−0.09	−0.03	−0.15	−0.04	−0.16	0.10	−0.14
Semantic	−0.04	0.05	−0.01	−0.23*	−0.06	0.04	0.02	−0.12	−0.12	−0.11	−0.11	−0.04	0.26*	0.03
Letter	−0.02	0.05	−0.01	0.25*	−0.06	0.04	0.02	−0.13	−0.13	−0.12	−0.13	−0.12	−0.10	0.04
Switches														
Free	−21*	−0.14	0.30*	−0.23*	−26*	0.22*	−0.01	−0.01	0.04	−0.04	−0.01	−0.06	0.10	−0.06
Semantic	−0.13	−0.11	0.09	0.10	0.01	0.24*	0.01	−0.09	−0.02	−0.07	−0.13	−0.11	0.10	−0.05
Letter	−0.16	−0.12	0.25*	−0.02	−0.19	0.20	0.14	−0.11	−0.08	−0.08	−0.12	−0.12	−0.06	−0.02

TMT = Trail-Making Test; RCTQ = Racing and Crowded Thoughts Questionnaire; RRS = Rumination Reponses Scale; YMRS = Young Mania Rating Scale; QIDS-C16 = Quick Inventory of Depressive Symptomatology - Clinician version; *p < 0.05.

Racing thoughts, assessed via the RCTQ, were associated with decreased cluster ratios and fewer words in the letter and semantic conditions. Specifically, ‘thought overexcitability’, i.e., distractibility, was linked to decreased verbal output in these task conditions. Higher brooding rumination scores, assessed via the RRS, were associated with fewer words in the free and the semantic VFT. Phonological and semantic cluster sizes were larger when response times in the Hayling task were longer. In the free VFT, switches decreased when word suppression in the Hayling task was impaired, and they increased with faster processing speed.

## Discussion

Word and error count in VFT could not clearly distinguish between mood episodes in BD. Indeed, only the depression group tended to produce fewer words compared to healthy controls and manic patients in the semantic VFT. These results are consistent with those reported by Raucher-Chéne *et al*.^8^, suggesting greater impairment in the semantic VFT in subgroups of patients with BD. By contrast, the process-oriented measures proved to better capture the combinatory, tangential, and sound-based speech found in mania^10^. As a matter of fact, in the free condition, manic patients switched more often between semantic subcategories than the healthy and depression groups, and their semantic cluster ratio was also reduced. However, these results were not observed when the tasks had retrieval rules, i.e. letter and semantic conditions. In the semantic condition, switches were increased in the manic group compared only to the depression and euthymic groups, but not the healthy one. Interestingly, we found larger task-discrepant phonological cluster sizes in manic patients compared to controls. Despite this, the error count was similar between groups. In the mixed depression group, the number of switches in the free VFT was also higher than those found in non-mixed depression, suggesting that subthreshold manic symptoms led to discrete structural speech anomalies.

Consistent with a previous study by Fossati *et al*.^28^ in unipolar depression, our results show reduced verbal output and switches in depressed patients, especially in the semantic VFT. Since this result was found in the semantic, but not the letter condition, this could be due either to a deterioration of the semantic system or to aberrant activation/inhibition processes within the semantic network^8^. Our results provide evidence against the storage deficit hypothesis, owing to the calculation of semantic cluster sizes which indexes semantic memory integrity. This index was not significantly different between groups, and vocabulary scores were equivalent between depression and healthy controls. Instead, in the semantic and free conditions, switching was decreased in the euthymic and the depression groups compared to the manic and healthy control groups, suggesting the existence of functional anomalies in the retrieval/access within the semantic system. Since results were similar in depression and euthymic groups, we argue that a trait-like impairment might be compensated for in the presence of manic symptoms^8,29,30^.

Interestingly, our results are the first to pinpoint switching abilities as affecting semantic fluency performance, and differently so among different types of mood episodes in BD. Like Fossati et al.’s findings^28^, switching was specifically correlated to measures of executive functions and psychomotor speed. Slower processing speed resulted in decreased switches in the semantic task, which might explain the results in depression and euthymia. As a whole, our results in bipolar depression are thus similar to those reported in unipolar depression^28^.

In contrast to depression, switches were increased in mania but also, to a lesser extent, in mixed depression. This was mainly observed in the free condition of VFT: subjects with mixed depression and mania, compared to depressed and euthymic patients, shifted from one discourse unit to the other at a faster rate, mimicking the flight of ideas characteristic of these states^10^. It is noteworthy however that increased switches did not amount to a greater number of words produced in the free VFT, despite the fact that cluster ratios and number of switches predicted the number of word output in all VFT. The stability of word output suggests that switches were increased at the expense of reduced semantic organization, as reflected by reduced cluster ratios in mania. All these results support the hypothesis of an abnormal access/retrieval within the semantic system might be involved in these results.

Indeed, a plausible explanation is that, in mania, there is a semantic overactivation during word retrieval. Clustering performance depends on the spread of semantic activation primed by each word generated^31^. Raucher-Chéné *et al*.^8^ had already put forward the possibility of a faster than usual spread of semantic activation to explain the ‘manic advantage’ in VFT. This hypothesis is consistent with our results in the free task. In addition, the results in VFT with retrieval rules are also supportive of this hypothesis rather than overall inhibition deficits. Deficient inhibition of unrelated words should have led to a greater amount of errors, smaller cluster ratios and increased switches in constrained VFT, but this was not the case in patients with manic symptoms relative to healthy controls. Correlation analyses did not support a straightforward role of inhibition deficits either, as clustering measures increased and switches decreased when inhibition was impaired in the Hayling task^32^ (see Supplementary Information).

The contrast between the results found in the free and the constrained VFT is striking, and the difference between these task conditions may be crucial to understand the mechanisms at play. In patients with manic symptoms, the unrestrictive nature of the free VFT may have enhanced diffuse semantic activation and favored the retrieval of more remotely associated words within the semantic network, which were not required to be inhibited in this task^26^. Hence, in the free condition, semantic overactivation was not detrimental to performance since subjects did not have to inhibit words unrelated to the task’s rules. A critical question is the role of distractibility. In the free task, it might have favored conceptual shifts, promoting the production of single words instead of clusters (i.e., reduced cluster ratio and increased switches) in patients with manic symptoms. However, distractibility might be detrimental to performance in tasks with retrieval rules, as suggested by the correlation between elevated racing thoughts, and its distractibility feature in particular (i.e., thought overexcitability), and decreased word output in restricted VFT. Yet again, this did not affect total word output in patients with manic symptoms. This, along with a similar error count, shows that patients with manic symptoms followed the tasks’ rules; that is, distractibility did not lead to irrelevant word production. Instead, the increased phonological cluster sizes in the semantic condition of VFT suggest that they are spontaneously more flexible. More specifically, when they had to produce animal names, manic subjects did so while rhyming and using other sound-based associations, akin to clanging, more than any other group. This is surprising given that phonological clustering is laborious and relies on executive functions^20,33^. However, enhanced executive functions in mania seems unlikely to explain our results, as executive performance was generally impaired in our manic group. Unrelated representations might rather be spontaneously activated through semantic spreading and subtend sound-based associations^34,35^. As emphasized above, semantic overactivation in mania might compensate for trait-like deficient word access/retrieval based on semantic cues.

It is noteworthy that manic symptoms, brooding rumination and racing thoughts were mainly correlated to decreased word output in patients, and most clinical symptoms were unrelated to switches or clustering measures. This suggests that self-report questionnaires fail to capture what is nonetheless clinically observed in the speech of patients, i.e. clanging and increased combinatory patterns^11^. This might be due to the fact that patients presenting with manic symptoms lack insight, hence underestimate their self-report of symptoms^36^. This makes the process-oriented VFT results all the more useful, as they provide a quantification of this clinical symptom, and improve the phenomenological models and scales aimed at evidencing enhanced spontaneous flexibility potentially involved in racing thoughts^14^. Importantly, clustering and switching abnormalities differentiated patients with manic symptoms, including the mixed depression group with few subthreshold – overlapping or not – hypomanic symptoms, from typical depression, suggesting that very few activation symptoms concurrent with depression might give rise to speech and thought abnormalities similar to those found in mania^25,37^.

Finally, we acknowledge the limitations of our study. The main one is the small sample of mixed manic patients. Given the small number of studies which have investigated language and cognition in mixed episodes of BD, results in mixed mania require to be confirmed by future studies. Second, we acknowledge the different lengths of the constrained and free VFT but it seems unlikely to explain the switching and clustering differences found here. Future studies may consider assessing the temporal pattern of process-oriented measures in BD, as it might provide further information regarding the cognitive mechanisms at play. For instance, these might be applied for within-cluster and non-clustered word intervals^20^ but also with focus on individual word production over the allotted VFT time, as retrieval during initial intervals (e.g., 15 s) might be associated with semi-automatic word retrieval, whereas later intervals are reflective of effortful word retrieval (e.g.^38–41^).

In sum, ours is the first study to capture thought and language abnormalities characteristic of mood episodes of BD, including mixed states, via process-oriented measures of VFT. This suggests that these measures may tackle structural and cognitive abnormalities that are not assessed simply by word or error count. Specifically, our results suggest that the increased combinatory nature of word output is subtended by a faster semantic spread of activation in patients with manic symptoms and might represent a compensatory mechanism for trait-like access/retrieval impairments found in euthymia^30^. Interestingly, these results were found in mania, but also in patients with mixed symptoms; increased switches were greater in mixed depression than non-mixed depression in the free VFT, mimicking the flight of ideas characteristic of mixed states.

## Methods

### Participants

Thirty-one healthy individuals aged 18–64 (M = 38.13, SD = 11.43) and 93 patients aged 18–64 (M = 42.70, SD = 12.83) with BD were recruited. Patients were recruited from inpatient and outpatient clinics at the University Hospital of Strasbourg, and fulfilled criteria for BD according to the DSM-IV-TR^42^. 50.5% patients had BD type 1, and 49.5% BD type 2. Healthy volunteers were recruited from the region by advertisement. They had no current or past personal history of psychiatric or neurological disorders nor did they have any first-degree relatives with psychosis or mood disorders. Patients with BD had no history of neurological disorder, ADHD, borderline personality disorder or substance use disorder within the last 12 months. Two patients were not taking any psychotropic medication at the time of the assessment. Of the remaining 91 patients, 39% were taking lithium, 44.1% were prescribed antiepileptic drugs, 40.9% were taking antipsychotics, 32.3% were on antidepressants, and 18% were taking benzodiazepines (27% of manic patients, 25% mixed manic patients, 20% mixed depression, 14% depressed, and 5% euthymia).

Detailed demographic data are presented in Table 3. Most patients in the manic and mixed manic groups presented with a hypomanic episode. Subjects provided written informed consent prior to inclusion in the study in accordance with the Declaration of Helsinki. This study was approved by the regional ethics committee of the East of France (CPP EST IV). All methods were performed in accordance with the aforementioned relevant guidelines and regulations.

**Table 3:** Means and standard deviations of demographic data of patients and controls.

	Mania	Mixed Mania	Mixed Depression	Depression	Euthymia	Controls	F/χ2	p
	n = 25	n = 12	n = 20	n = 17	n = 20	n = 31		
Age	39.52 (14.9)	42.67 (9.23)	42.9 (11.89)	45.29 (12.84)	43.7 (13.88)	38.12 (11.52)	F(5,119) = 1.05	0.39
Sex (F/M)	14/11	7/5	17/3	10/7	10/10	23/8	χ2 (5, n = 124) = 7.38	0.19
Years of education	14.2 (2.04)	12.75 (2.6)	14.35 (1.95)	14.95(2.67)	15.05 (2.33)	14.1 (2.37)	F(5,119) = 1.77	0.12
Illness duration (months)	152.52 (125.16)	131 (104.77)	196.72 (153.10)	209.5 (203.47)	193.54 (145.92)	/	F(4,77) < 1	0.64
Number of hospitalizations	2.22 (2.68)	1.64 (1.29)	3.94 (3.82)	2.75 (2.11)	3.19 (2.9)	/	F(4,77) = 1.5	0.21
Past depressive episodes (number)	5.24 (6.20)	7 (6.28)	6.06 (3.49)	5.6 (3.37)	4 (3.14)	/	F(4,69) < 1	0.64
Past manic episodes (number)	4.30 (4.37)	3.89 (3.98)	4 (3.83)	4.13 (3.58)	3.93 (3.02)	/	F(4,71) < 1	0.99
Episodes with psychotic features	0.93 (2.09)	1.33 (1.61)	0.68 (1.67)	0.62 (1.42)	1.45 (2.01)	/	F(4,77) < 1	0.48
YMRS	12.56 (3.59)	8.67 (2.31)	4 (1)	0.65 (0.86)	1.15 (1.63)	0.61 (0.88)	F(5,118) = 138.9	<0.001
QIDS-C16	2.4 (1.55)	9.58 (2.78)	12.79 (3.07)	11.88 (3.22)	1.45 (1.57)	0.87 (1.20)	F(5,118) = 130.17	<0.001

YMRS = Young Mania Rating Scale; QIDS-C16 = Quick Inventory of Depressive Symptomatology-Clinician version; significant results are presented in italics.

### Materials and procedures

Patients were considered to be in a predominantly depressive or manic/(hypo)manic episode if they fulfilled the DSM-IV-TR criteria for either episode^42^. Comorbidities were assessed by senior psychiatrists using the DSM-IV-TR criteria^42^. Patients had no history of neurological disorder, ADHD, substance use disorder within the last 12 months or borderline personality disorder. Prior to the neuropsychological assessment, mania and depression symptoms were assessed with the Young Mania Rating Scale (YMRS)^43^ and the Quick Inventory of Depressive Symptomatology–Clinician-Rated Version (QIDS-C16)^44^. A YMRS score > 5 was considered reflective of hypomania^45^ and a QIDS-C16 score > 5 was reflective of depression^43^. A mixed manic/hypomanic state was diagnosed if manic and depressive symptoms were above the cut-off^46^. Mixed depression was diagnosed when scores were above the threshold for depressive symptoms (QIDS-C16 score > 5), and co-occurred with mild hypomanic symptoms (YMRS score > 2 and <6)^47^. Euthymia was defined by scores below the threshold in both the YMRS and the QIDS-C16, reflecting the absence of a significant mood episode. Psychotic features were not part of the affective episodes at the time of testing, as assessed by the YMRS item 8 referring to thought content.

Participants then fulfilled two self-rated questionnaires, the Racing and Crowded Thoughts Questionnaire (RCTQ)^48^ assessing three facets of racing thoughts – i.e., ‘thought overactivation’, its ‘burden’, and ‘overexcitability’ features–, and the Ruminative Response Scale State-version (RRS-S)^49^ assessing ‘brooding’ and ‘reflection’ rumination. They were also administered a battery of neuropsychological tests, including three VFT.

#### Neuropsychological assessment

Neuropsychological assessment included measures of processing speed and attention switching – i.e., the Trail Making Test (TMT-A & B)^50^ and the digit-symbol subtest of the Wechsler Adult Intelligence Scale—Third Edition (WAIS-III)^51^. Semantic inhibition was assessed via the Hayling test^52^, which requires participants to orally complete a set of 15 sentences, whose last word is missing, with semantically-unrelated ending words. Both response times and errors were recorded. Working memory was assessed via the digit-span task^50^. The Vocabulary Subtest of the WAIS-III assessed subjects’ lexico-semantic abilities and vocabulary size^53^ and the French National Adult Reading Test^54^ measured their premorbid intellectual functioning^32^.

#### Verbal fluency tasks

The three conditions of the verbal fluency task were administered in a fixed order, starting with the most unrestrictive condition^55^: the free, the letter and the semantic conditions. In the free fluency trial, participants were asked to produce as many words as possible, with their eyes closed, during 150s^56^. In the letter fluency condition, subjects were asked to produce as many words as possible starting with the letter ‘p’, with the exception of proper nouns, during 120 s. Words starting with a letter other than p and proper nouns were counted as errors^55^. In the semantic VFT, participants had to produce as many animal nouns as possible, during 120 s. Words belonging to different semantic categories were counted as errors^55^.

Scoring procedure: Participants’ oral production was recorded using the Audacity© software. Verbatim output was transcribed by French-speaking psychology undergraduates who were blind to the diagnostic status of the participants.

In addition to total word and error count, semantic and phonological cluster ratios (i.e., number of clusters/number of words), mean cluster sizes, and the raw number of switches were calculated for the three VFT. Two independent raters (graduate-level psychologists) blind to the diagnostic status of participants scored the verbal fluency protocols. Semantic and phonemic relatedness were assessed in the three conditions of the task; this combined procedure allows for the identification of both task-consistent and task-discrepant clustering^20^. Additionally, it allows for the calculation of cluster ratios, i.e., the number of clusters/number of words, and mean cluster size, i.e., total number of words in clusters beginning with the second word divided by the number of clusters produced^21,22^. The former indexes output organization whereas the latter indexes the integrity of the lexico-semantic store. Cluster sizes of zero were not considered in the analysis^19^. Semantic clusters were defined as a group of at least two serially produced words related categorically (e.g., fruits), or contextually (e.g., animals that live in the forest). Synonyms and antonyms, but also superordinates, were considered as being related. Phonemic clusters were defined as groupings of at least two serially produced words sharing the first two phonemes (e.g., plot and plight), sharing a syllable (e.g., propensity and pen), rhyming (e.g., daughter and water), differing only by a vowel sound (e.g., pin, pen), as well as homonyms (e.g., sum and some)^17^. Switches were defined by shifts from a cluster to another cluster, but also from a cluster to a word, or from a word to another word^17,19^. Intra-class correlations between scoring of the two raters revealed excellent interrater reliability for both semantic and phonemic clustering in the letter (r = 0.86, r = 0.95, respectively), semantic (r = 0.88, r = 0.93, respectively) and free (r = 0.93, r = 0. 95, respectively) conditions.

### Statistical analyses

Analyses were undertaken using the Statistica® software. Because data were normally distributed for the whole sample of participants (Kolmogorov-Smirnov test, p > 0.10), for each scoring criterion and condition of the task, we conducted a one-way ANCOVA with group as a between-group variable and the following covariates, given their potential effect on cognitive performance^6,57^: (i) number of hospitalizations, (ii) lithium dosage, and (iii) equivalent dosage of antipsychotic drugs. Of note, results were similar when the covariates were not entered in the analyses. Based on our a priori hypotheses, planned comparisons between groups were performed using the false discovery rate (FDR)^58^ method of alpha level adjustment for multiple comparisons; statistical significance was set at 0.05 (two-sided tests). Specifically, planned comparisons were conducted between manic patients’ performance compared to depression, euthymia and controls, on the one hand, and depression versus mixed depression, on the other hand. Given the scarce literature on language and cognition in mixed mania, we did not have a priori hypotheses regarding this group and planned comparisons were not performed for this group in particular. Results were similar when subjects taking benzodiazepines at the time of the assessment were removed from the analyses, hence for the sake of simplicity we will present the results averaged over all subjects. Only significant planned comparisons were reported in the results section for greater readability. Correlation analyses, using Pearson’s coefficient, were performed in the whole sample of patients and in patients with manic symptoms alone between the verbal fluency measures, neuropsychological measures, and clinical symptoms. To investigate the relationship between process-oriented measures (clustering and switching) and word output in the three VFT, we conducted multiple regression analyses on the word output within the patient sample. Five predictors – i.e., semantic cluster size, phonological cluster size, ratio of semantic clusters, ratio of phonological clusters, and number of switches –, were simultaneously entered into the model. Statistical significance was set at 0.05.

## Supplementary information

Supplementary results and discussion

## Data Availability

Data can be made available upon request to authors.
