# Supplementary material for: Thought and language disturbance in bipolar disorder quantified via process-oriented verbal fluency measures
Source: Sci Rep. 2019 Oct 3;9:14282. doi: 10.1038/s41598-019-50818-5 (PMC6776521; doi:10.1038/s41598-019-50818-5)
Supplement: Supplementary file 1 — Supplementary results and discussion [file 41598_2019_50818_MOESM1_ESM.docx]

**Supplementary information**

**Thought and language disturbance in bipolar disorder quantified via process-oriented verbal fluency measures**

**Luisa Weiner^a,b*^, Nadège Doignon-Camus^a^, Gilles Bertschy^a,b,e^, Anne Giersch^a^**

^a^ INSERM U1114, Strasbourg, France

^b^ Psychiatry Department, University Hospital of Strasbourg, France

^e^ Translational Medicine Federation, University of Strasbourg, France

*Corresponding author:

Luisa Weiner, INSERM1114 - Department of Psychiatry - University Hospital of Strasbourg - 1 place de l’hôpital- 67000 Strasbourg

Email: [Luisa.weiner@chru-strasbourg.fr](mailto:Luisa.weiner@chru-strasbourg.fr), phone number: + 0033 388116511

**2– Results**

| **Table 4:** Correlations between verbal fluency and neuropsychological and clinical measures in patients with manic symptoms (n = 53) | | | | | | | | | | | | | | | | |
| --- | --- | --- | --- | --- | --- | --- | --- | --- | --- | --- | --- | --- | --- | --- | --- | --- |
|  | **TMT - A** | | **TMT-B** | **Digit-span** | **Hayling time** | **Hayling errors** | **Digit-Symbol** | **Vocabulary** | **RCTQ** | **RCTQ** | **RCTQ** | **RCTQ** | **RRS** | **YMRS** | **QIDS-C16** |  |
| **Instruments** |  |  |  |  |  |  |  |  | **total** | **overactivation** | **burden** | **overexcitability** | **brooding** |  |  |  |
| **Number of words** |  | |  |  |  |  |  |  |  |  |  |  |  |  |  |  |
| Free | -.26 | | -.28* | .57* | -.23 | -.24 | .23 | .18 | -.28* | -.21 | -.28* | -.25 | -.41* | .07 | -.17 |  |
| Semantic | -.32* | | -.42* | .49* | -.17 | -.10 | .38* | .37* | -.34* | -.25 | -.29* | -.37* | -.33* | .08 | -.18 |  |
| Letter | -.27* | | -.38* | .59* | -.39* | -.32* | .40* | .34* | -.16 | -.11 | -.11 | -.17 | -.19 | -.14 | -.04 |  |
| **Semantic cluster ratio** |  | |  |  |  |  |  |  |  |  |  |  |  |  |  |  |
| Free | .02 | | -.12 | .13 | .18 | .08 | .03 | -.08 | -.03 | .01 | .01 | -.04 | -.15 | -.15 | -.02 |  |
| Semantic | .03 | | -.09 | .12 | .09 | .12 | .09 | .03 | -.10 | .03 | -.05 | -.20 | -.15 | .13 | -.10 |  |
| Letter | -.26 | | -.28* | .32* | .08 | .01 | .25 | .21 | -.17 | -.18 | -.11 | -.20 | -.20 | -.10 | .01 |  |
| **Semantic cluster size** |  | |  |  |  |  |  |  |  |  |  |  |  |  |  |  |
| Free | -.04 | | -.02 | .02 | -.14 | -.08 | -.10 | .04 | -.11 | -.12 | -.10 | -.10 | -.08 | .27* | -.05 |  |
| Semantic | -.03 | | -.09 | .14 | .08 | .09 | .10 | .06 | -.11 | .03 | -.05 | -.20 | -.15 | .11 | -.08 |  |
| Letter | -.08 | | .01 | .04 | -.19 | -.02 | .16 | .22 | -.12 | -.13 | -.09 | -.05 | -.14 | -.17 | .16 |  |
| **Phonological cluster ratio** | | |  |  |  |  |  |  |  |  |  |  |  |  |  |  |
| Free | | .14 | .16 | .16 | -.12 | .04 | -.19 | .02 | -.07 | .01 | -.16 | -.01 | -.19 | .21 | -.13 |  |
| Semantic | | .07 | .10 | .08 | .02 | .05 | -.01 | .04 | -.32* | -.24 | -.35* | -.25 | -.28 | .09 | -.14 |  |
| Letter | | .11 | -.01 | .05 | .02 | .08 | -.10 | .09 | -.26 | -.30* | -.16 | -.25 | -.12 | -.09 | .07 |  |
| **Phonological cluster size** | |  |  |  |  |  |  |  |  |  |  |  |  |  |  |  |
| Free | | -.08 | -.04 | .23 | -.12 | -.12 | -.01 | .05 | -.20 | -.01 | -.15 | -.01 | -.20 | .29* | -.24 |  |
| Semantic | | -.14 | -.02 | .13 | -.28* | -.13 | .01 | .17 | -.06 | -.02 | -.11 | -.01 | -.07 | .07 | -.20 |  |
| Letter | | .18 | .04 | .01 | .02 | .03 | -.05 | -.01 | .01 | -.07 | -.06 | -.01 | -.12 | '-.21 | -.16 |  |
| **Switches** | |  |  |  |  |  |  |  |  |  |  |  |  |  |  |  |
| Free | | -10 | -.18 | .36* | -.20 | -.33* | .13 | .15 | -.18 | -.14 | -.21 | -.15 | -.22 | -.10 | -.16 |  |
| Semantic | | -.14 | -.21 | .19 | -.02 | .06 | .29* | .05 | -.11 | -.01 | -.07 | -.18 | -.17 | .14 | -.17 |  |
| Letter | | -.19 | -.29* | .57* | -.44* | -.28* | .28* | .33* | -.02 | -.06 | .01 | -.06 | -.21 | -.04 | -.10 |  |
| Legend: TMT = Trail-Making Test;RCTQ = Racing and Crowded Thoughts Questionnaire; RRS = Rumination Reponses Scale; YMRS = Young Mania Rating Scale; QIDS-C16 = Quick Inventory of Depressive Symptomatology - Clinician version; * p<.05 | | | | | | | | | | | | | | | |  |

**3- Discussion**

In the Hayling task, subjects have to inhibit words that complete a series of sentences; hence, inhibition difficulties lead to the production of semantically-related words. In VFT, semantic-relatedness, i.e., spreading of activation, is reflected by cluster sizes^17^, and this is probably why cluster sizes were larger when inhibition was more deficient in the Hayling task.
